# Improved Right Ventricular Performance with Increased Tricuspid Annular Excursion in Athlete’s Heart

**DOI:** 10.3389/fcvm.2015.00008

**Published:** 2015-04-30

**Authors:** Wei Zha, Chun G. Schiros, Gautam Reddy, Wei Feng, Thomas S. Denney, Steven G. Lloyd, Louis J. Dell’Italia, Himanshu Gupta

**Affiliations:** ^1^Department of Medical Physics, University of Wisconsin-Madison, Madison, WI, USA; ^2^Department of Medicine, University of Alabama at Birmingham, Birmingham, AL, USA; ^3^Department of Biomedical Engineering, Wayne State University, Detroit, MI, USA; ^4^Department of Electrical and Computer Engineering, Auburn University, Auburn, AL, USA; ^5^Birmingham Veteran Affairs Medical Center, Birmingham, AL, USA

**Keywords:** cardiac magnetic resonance imaging, right ventricular function, tricuspid annulus displacement, interventricular septal remodeling, marathon runners, right ventricle strain, mitral regurgitation

## Abstract

**Background:**

Marathon runners (MTH) and patients with mitral regurgitation (MR) exhibit left ventricular (LV) overload, and LV geometric changes in these groups have been reported. In this study, right ventricular (RV) adaptation to chronic volume overload was evaluated in MTH and MR and normal controls together with interventricular septal remodeling and tricuspid annulus (TA) motion.

**Methods:**

A total of 60 age-matched subjects (including 19 MTH, 17 isolated chronic compensated MR patients, and 24 normal subjects) underwent conventional cine and tagged cardiac magnetic resonance imaging. Myocardial strain and curvature were computed on the interventricular septum and RV free wall. A dual-propagation technique was applied to construct RV volume-time curves for a single cardiac cycle. Similarly, the TA was tracked throughout the cardiac cycle to create displacement over time curve.

**Results:**

Septal curvature was significantly lower in MTH and MR compared to controls. No significant differences in RV free-wall strain or RV ejection fraction were noted among the three groups. However, longitudinal TA excursion was significantly higher in MTH compared to controls (*p* = 0.0061). The peak late diastolic TA velocity in MR was significantly faster than MTH (*p* = 0.0031) and controls (*p* = 0.020).

**Conclusion:**

Increased TA kinetics allows for improved RV performance in MTH. Septal remodeling was observed in both MR and MTH, therefore a direct relationship of septal remodeling to TA kinetics in athlete’s heart could not be elucidated in this study.

## Introduction

The left ventricle undergoes remodeling in response to sustained changes in left ventricular (LV) pressure or volume load. In previous work, we used cardiac magnetic resonance (CMR) imaging to study LV changes in marathon runners (MTH), where the dilated left ventricle maintains an ellipsoid shape, and in mitral regurgitation (MR), where the dilated left ventricle becomes more spherical (1). Thus, the left ventricle responds differently to physiologic and pathologic chronic volume loading conditions. The stresses that lead to this remodeling are also transmitted to the right ventricle. The interventricular septum (IVS) allows for direct interaction of left and right ventricles and hence transmits systolic and diastolic forces between the ventricles. Previous studies (2–5) have shown that the deleterious effects of LV dilation in severe chronic organic MR on the structure and function of the right ventricle, leading to compression, flattening, and the consequential impairment of right ventricular (RV) systolic function. However, the influence of IVS remodeling in compensated MR on RV function has not been well studied. Furthermore, the relationship of differential LV remodeling due to physiologic versus pathologic LV volume overload to RV function has not been well described. In this study, three-dimensional (3D) geometric analysis and Lagrangian strain computation were used to define IVS remodeling and mechanics in compensated MR and MTH. We then evaluated the relationship of RV functional indices to IVS remodeling in these conditions. We hypothesized that maintenance of favorable LV and IVS geometry in physiologic LV overload noted in MTH would have favorable impact on RV function compared to pathologic LV volume overload in MR.

## Materials and Methods

This study includes the same cohort of 60 subjects as previously described (1), which included 19 MTH (mean age 39 ± 10 years, 47% female), 17 patients with degenerative isolated MR (mean age 46 ± 5, 53% female), and 24 controls (mean age 45 ± 8 years, 50% female). Chronic isolated MR was defined as at least moderate severity with LV EF >60% based on echocardiographic/Doppler examination in the absence of symptoms or obstructive coronary artery diseases determined by exercise testing with nuclear perfusion. No MR patient had a history of hypertension or was taking any medication at the time of study. The study protocol was approved by the Institutional Review boards of the University of Alabama at Birmingham and Auburn University. All participants gave written informed consent.

Conventional cine MRI was acquired on a 1.5-T magnetic resonance scanner (GE Signa, Milwaukee, WI, USA) to obtain standard (2-, 3-, and 4-chamber long-axis and serial parallel short-axis) views using prospective electrocardiographically gated, breath-hold, steady state free-precession technique with following scan parameters: slice thickness = 8 mm, zero interslice gap, field of view = 40 cm × 40 cm, scan matrix = 256 × 128, flip angle = 45°, repetition/echo times = 3.8/1.6 ms. Twenty cardiac phases were reconstructed with 8–10 views per segment.

Tagged CMR was acquired with the same slice prescription as the cine acquisition. Grid tags were applied to short-axis views and stripe tagging to long-axis views using the spatial modulation of magnetization encoding method with the following scan parameters: prospective ECG gating, slice thickness = 8 mm, zero interslice gap, field of view = 40 cm × 40 cm, scan matrix = 256 × 128, flip angle = 10°, repetition/echo times = 8.0/4.2 ms, views per segment = 8–10, tag spacing = 7 mm, 20 reconstructed cardiac phases.

### RV volumetric and tricuspid annular kinetics analysis

**Figure 1:**
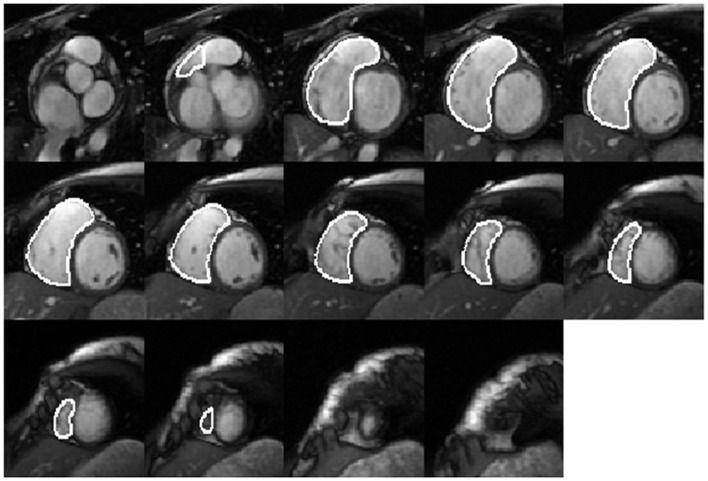
**Manually drawn RV endocardial contours at end diastole in a short-axis view**.

**Figure 2:**
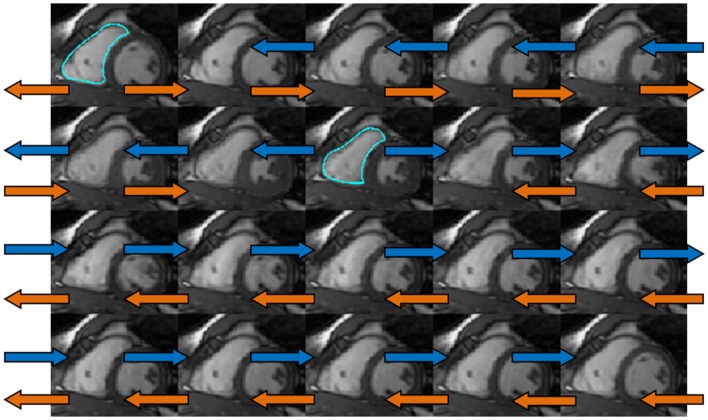
**The dual-propagation diagram on a mid-ventricular short-axis slice**. The orange and blue arrows indicated the propagation using end diastolic (ED) and end systolic (ES) contours as the templates. These two sets of propagated contours were then combined via a weighted least-square fit to obtain the dual-propagated contour at each timeframe other than ED and ES.

**Figure 3:**
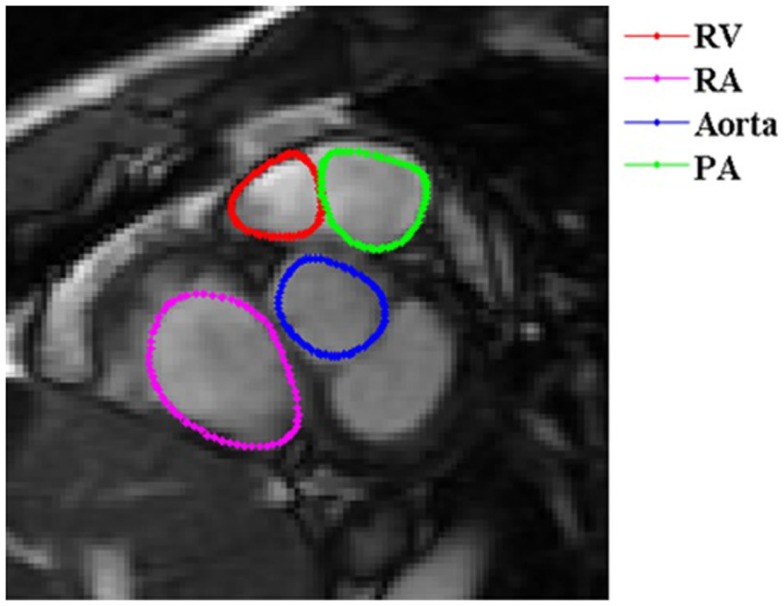
**Short-axis view of a basal RV slice**. In basal slices of short-axis views, the right ventricle (red) coexists with the right atrium (RA) (magenta), aorta (blue), and pulmonary artery (PA) (green).

Right ventricular endocardial contours at end-diastole (ED) (Figure 1) and end-systole (ES) were manually drawn as closed contours between the tricuspid annulus (TA) and RV apex from the short-axis views. These contours were then automatically propagated to all the other frames in the acquisition using a dual-contour propagation algorithm (6) as illustrated in Figure 2. Accurate RV segmentation in basal slices is difficult due to TA motion and partial volume effects as depicted in Figure 3. In conventional short-axis images, the RV inflow and outflow tracts are obscure. The borders among right ventricle, right atrium, and pulmonary artery were identified by viewing long-axis series and their slice projections simultaneously. To further account for this, a user-selected landmark point was specified at the TA on the RV lateral wall in both ED and ES frames of a four-chamber slice. Then, a non-rigid registration algorithm, similar to the one used in Ref. (6) for tracking mitral annular motion, was used to track this point through the cardiac cycle to form a displacement versus time curve. The displacement of this point perpendicular to the short-axis image plane was used to determine the fraction of basal short-axis slice that contributed to RV volume in that particular phase of the cardiac cycle. The validation of this propagation method on right ventricle is presented in the Data Sheet 1 in Supplementary Material.

Once the volumes were computed at each time frame, a RV volume-time curve (VTC) was constructed and differentiated with respect to time. Early diastole and late diastole were defined as the first and second halves, respectively, of the diastolic interval. RV peak ejection rate (PER) was defined as the maximum negative time derivative during the systolic interval. RV early peak filling rate (ePFR) and atrial peak filling rate (aPFR) were defined as the maximum derivative during the early and late diastole. RV e/a ratio was computed as RVePFR over RVaPFR.

### Interventricular septal regional analysis

**Figure 4:**
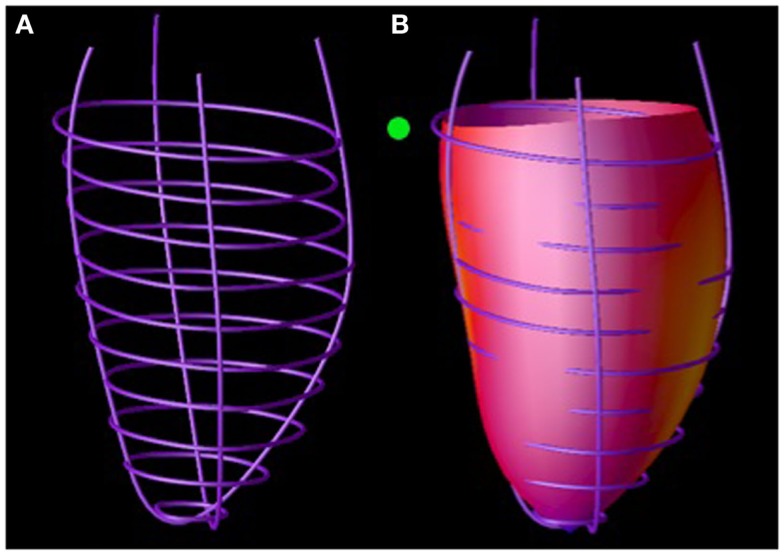
**Septal wall curvature computation**. LV endocardial contours manually traced on magnetic resonance images were positioned in 3D **(A)**. A surface was fit to these contours **(B)**. Septal wall curvature was computed from the surface curvature at the septal wall (denoted by the green ball).

Three-dimensional (3D) IVS geometric parameters were measured from LV endocardial and epicardial contours manually traced on cine images acquired near end-diastole and end-systole as described previously (1, 7–15). The contours were traced to exclude papillary muscles. The contour data were then transformed to a coordinate system aligned along the long-axis of the left ventricle (Figure 4A) and converted to a prolate spheroidal coordinate system as described in Ref. (16). Cubic B-spline surfaces with 12 control points in the circumferential (θ) direction and 10 control points in the longitudinal direction (μ) were fit to the λ coordinates of the LV endocardial and epicardial contours (Figure 4B) for each time frame. The fit used the smoothing term described in Ref. (16), with α = 0, β = 0, and γ = 0.1.

Three-dimensional (3D) endocardial surface curvatures were computed using standard formulas (17) at the septal wall segments defined in Ref. (18) (Figure 4B). Three-dimensional wall thickness was computed at the same segments by measuring the distance from a point on the LV epicardial surface to the closest point on the LV epicardial surface along a line perpendicular to the LV epicardial surface. The radius of curvature to wall thickness ratio (R/T) was computed by computing the reciprocal of the product of the endocardial circumferential curvature and wall thickness.

Three-dimensional LV strains were measured from tagged images at ES. Tag lines were tracked using a tag extraction technique (19) and edited by an expert user if necessary. 3D deformation and Lagrangian strain were computed by fitting a prolate spheroidal B-spline deformation model to the tag line data (20). The maximum principle strain was approximately aligned in the radial direction and is the maximum thickening strain. The maximum shortening strain was approximately aligned with circumferential direction and is the maximum contraction strain.

Interventricular septum geometric and strain parameters at the base and mid-ventricular levels were computed by averaging the LV anteroseptal and inferoseptal segments at each level. At the distal level, septal parameters were measured from the LV apical septal segment.

### RV curvature and strain measurements

Three-dimensional RV geometric parameters were measured using the techniques described above modified for the right ventricle. The RV lateral wall was divided into eight segments: three at base, three at mid, and two at apical level. 2D RV strains were calculated using harmonic phase (HARP) analysis (21). 2D RV mid-ventricular maximum shortening is the minimum principal strain averaged over the RV lateral wall segments at the mid-ventricular level.

### Statistical analysis

One-way analysis of variance was used to compare groups for continuous and categorical variables as performed for left ventricle analysis among three subject groups. Homogeneity of variance was tested using Levene’s test. Model normality assumption was checked and if it was violated, appropriate data transformation was conducted. Tukey–Kramer procedure was performed to control the pairwise comparisons among the groups jointly in order to avoid erroneous type I error rate inflation.

Data are presented as mean ± SD. A *p* value <0.05 was considered statistically significant. For repeated measures, a *p* < 0.01 was considered statistically significant to account for correlations among parameters and locations of measurements (Table 2). All statistical analyses were performed using SAS version 9.2 (SAS Institute Inc., NC, USA).

## Results

**Figure 5:**
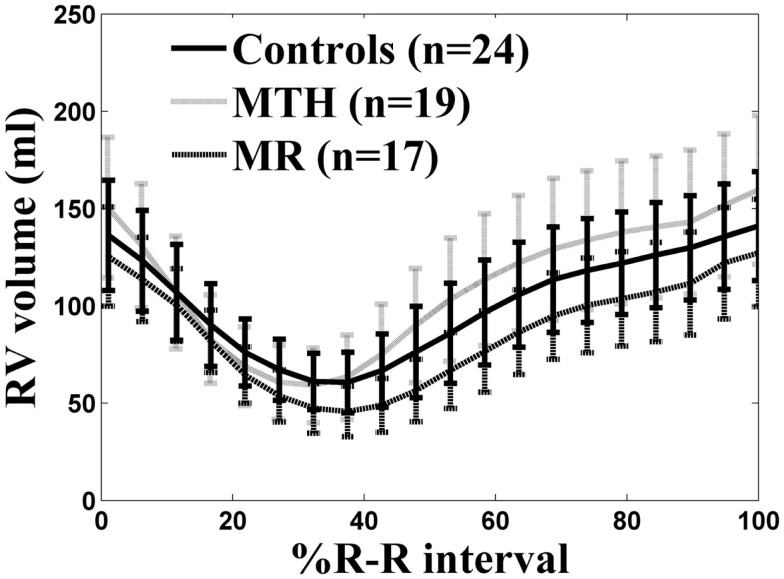
**RV volume-time curves for controls (solid line), marathon runners (gray line), and mitral regurgitation patients (dashed line)**. The solid, gray, dashed lines are the average RV volume-time curves of three groups. The error bars represent the SD at each measured time point.

As previously described (1), all participants had normal LVEF >55%. The RVEDV, RVESV, and stroke volume indices (normalized to body surface area) in MTH were significantly higher than MR or controls (Table 1). Mitral regurgitant volume and fraction of the MR patients was 29 ± 17 ml and 25 ± 14%. The RV mass index was significantly higher in MTH compared to MR or controls both with *p* < 0.0001(Table 1). This indicates that the increased demands on the right ventricle in MTH elicit greater remodeling effects than in MR. There are distinct differences in the overall RVVTCs of MTH and MR compared to controls as depicted in Figure 5. The absolute RVPER was significantly higher in MTH compared to controls (*p* = 0.044). However, once normalized to RVEDV, it was no longer different among the groups. Although there were differences in the RVePFR and RV e/a ratio between MTH and MR, they did not reach statistical significance.

**Table 1:** **RV free-wall geometry, strain, and ejection and filling rate**.

	Controls	MTH	MR
RV end-diastolic volume index (ml/m^2^)	72 ± 11	104 ± 13*	78 ± 15^†^
RV end-systolic volume index (ml/m^2^)	34 ± 8	47 ± 8*	35 ± 8^†^
RV stroke volume index (ml/m^2^)	39 ± 8	58 ± 8*	43 ± 10^†^
RV ejection fraction (%)	54 ± 8	55 ± 5	55 ± 7
RV mass index (g/m^2^)	15.76 ± 3.96	23.73 ± 5.79*	14.56 ± 3.23^†^
RV ED mid lateral 3D curvature (1/cm)	0.38 ± 0.10	0.44 ± 0.12	0.44 ± 0.09
RV ES mid lateral 3D curvature (1/cm)	0.69 ± 0.20	0.56 ± 0.18	0.62 ± 0.18
RV 2D lateral wall maximum shortening (%)	20.17 ± 2.50	20.21 ± 1.81	19.94 ± 2.05
RVPER (ml/s)	366.43 ± 100.96	444.34 ± 115.90*	378.96 ± 90.06
RVPER in RVEDV/s	2.59 ± 0.49	2.81 ± 0.51	3.00 ± 0.63
RVePFR in RVEDV/s	1.96 ± 0.50	1.99 ± 0.41	2.19 ± 0.45
RVePFR (ml/s)	274.18 ± 74.84	312.98 ± 86.55	275.49 ± 65.60
RVaPFR (ml/s)	153.77 ± 82.57	164.49 ± 105.83	179.22 ± 62.36
RVaPFR in RVEDV/s	1.11 ± 0.58	1.06 ± 0.73	1.42 ± 0.50
RV e/a ratio (−)	2.27 ± 1.21	2.53 ± 1.40	1.66 ± 0.46

*Values are expressed as mean ± SD*.

*p < 0.05 vs. controls.

*^†^*p* < 0.05 vs. marathon runners*.

*RVPER, right ventricular peak ejection rate; RVePFR, right ventricular peak early filling rate; RVaPFR, right ventricular peak atrial filling rate; ED, end diastolic; ES, end systolic; Mid, mid-ventricular*.

**Figure 6:**
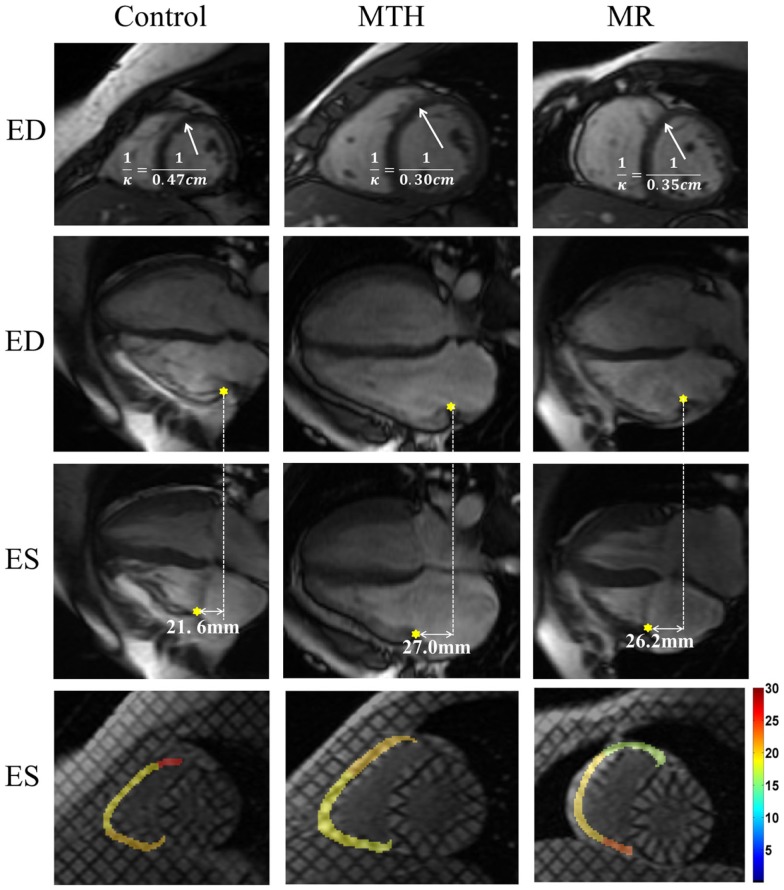
**End diastolic septal mid-ventricular curvature and RV tricuspid annulus displacement and strain map for a control, marathon runner, and mitral regurgitation patient**. The marathon runner and the patient with MR had significantly increased septal mid curvature compared to the control. The tricuspid annulus displaced more in the marathon runner than it in the control. The RV lateral wall maximum shortening strain was similar in three groups.

The IVS radius-thickness ratio in ED was increased at distal level in MR versus controls (Table 2). The IVS circumferential curvature was significantly reduced at all three levels in MTH during both ED (*p* ≤ 0.0007 at all levels) and ES (*p* ≤ 0.0064 at all levels), whereas it was decreased at the basal (*p* = 0.0013) and mid (*p* = 0.0061) levels in ED and at the distal (*p* = 0.005) level in ES in MR (Table 2; Figure 6). Thus, the IVS is flatter in both MTH and MR compared to controls. The IVS wall thickness, maximum shortening, and maximum principle strain in the IVS were similar in the three groups at all levels. RV free-wall maximum shortening strains were also similar among three groups (Table 1). Despite similar thickening and strains, the mid and distal septal ES torsion were significantly less in MTH compared to controls (*p* = 0.0065 at mid; *p* = 0.0098 at distal). However, the local torsion shear angle in MTH was similar to both MR and controls.

**Table 2:** **Septal local geometry and strain**.

	Base			Mid			Distal		
	Controls	MTH	MR	Controls	MTH	MR	Controls	MTH	MR
Septal ED circ. curv. (1/cm)	0.36 ± 0.03	0.29 ± 0.04*	0.31 ± 0.04*	0.39 ± 0.03	0.33 ± 0.04*	0.35 ± 0.04*	0.48 ± 0.04	0.41 ± 0.06*	0.44 ± 0.06
Septal ES circ. curv. (1/cm)	0.52 ± 0.04	0.47 ± 0.06*	0.48 ± 0.04	0.57 ± 0.06	0.48 ± 0.08*	0.49 ± 0.05	0.73 ± 0.11	0.61 ± 0.09*	0.64 ± 0.06*
Septal ED wall thickness (cm)	0.83 ± 0.15	0.86 ± 0.08	0.82 ± 0.12	0.75 ± 0.18	0.74 ± 0.09	0.69 ± 0.11	0.60 ± 0.15	0.56 ± 0.09	0.50 ± 0.07
Septal ES wall thickness (cm)	1.17 ± 0.18	1.22 ± 0.12	1.22 ± 0.18	1.17 ± 0.20	1.20 ± 0.15	1.14 ± 0.14	1.02 ± 0.25	0.99 ± 0.21	0.87 ± 0.12
Septal ED RT ratio (−)	3.44 ± 0.73	4.01 ± 0.61	3.92 ± 0.59	3.68 ± 0.98	4.27 ± 0.65	4.40 ± 0.95	3.74 ± 1.19	4.45 ± 0.75	4.67 ± 0.81*
Septal ES RT ratio (−)	1.66 ± 0.43	1.73 ± 0.23	1.71 ± 0.29	1.59 ± 0.41	1.78 ± 0.26	1.82 ± 0.28	1.46 ± 0.53	1.74 ± 0.30	1.81 ± 0.27
Septal wall thickening (%)	42.05 ± 15.44	43.88 ± 7.95	50.08 ± 11.40	59.06 ± 22.76	63.87 ± 13.50	66.70 ± 16.42	71.37 ± 29.26	77.08 ± 20.91	76.28 ± 25.36
Septal ES 3D maximum shortening (%)	19.12 ± 2.61	17.86 ± 2.23	19.96 ± 2.98	18.62 ± 2.70	17.74 ± 3.12	18.45 ± 2.20	21.67 ± 5.08	19.26 ± 3.13	20.24 ± 3.36
Septal ES maximum 3D principle strain (%)	15.87 ± 12.53	13.87 ± 9.79	14.19 ± 8.12	21.24 ± 9.52	26.66 ± 10.62	24.23 ± 10.92	16.59 ± 12.19	23.85 ± 17.29	22.74 ± 21.27
Septal ES twist angle (°)	3.37 ± 1.10	2.94 ± 0.98	3.29 ± 0.91	8.46 ± 2.10	6.98 ± 2.01	8.11 ± 1.77	13.42 ± 3.56	11.45 ± 2.92	12.13 ± 3.00
Septal ES torsion (°/cm)	3.76 ± 1.12	3.04 ± 1.21	3.64 ± 1.03	2.93 ± 0.69	2.24 ± 0.80*	2.75 ± 0.59	2.86 ± 0.73	2.22 ± 0.72*	2.54 ± 0.60
Septal ES torsion shear angle (°)	13.17 ± 4.08	11.94 ± 4.20	13.67 ± 4.01	8.95 ± 2.24	7.64 ± 2.27	9.10 ± 1.98	9.17 ± 2.40	7.97 ± 2.09	8.74 ± 2.08

*Values are expressed as mean ± SD*.

*p < 0.01 vs. controls.

*ED, end diastolic; ES, end systolic; circ. curv., circumferential curvature*.

**Figure 7:**
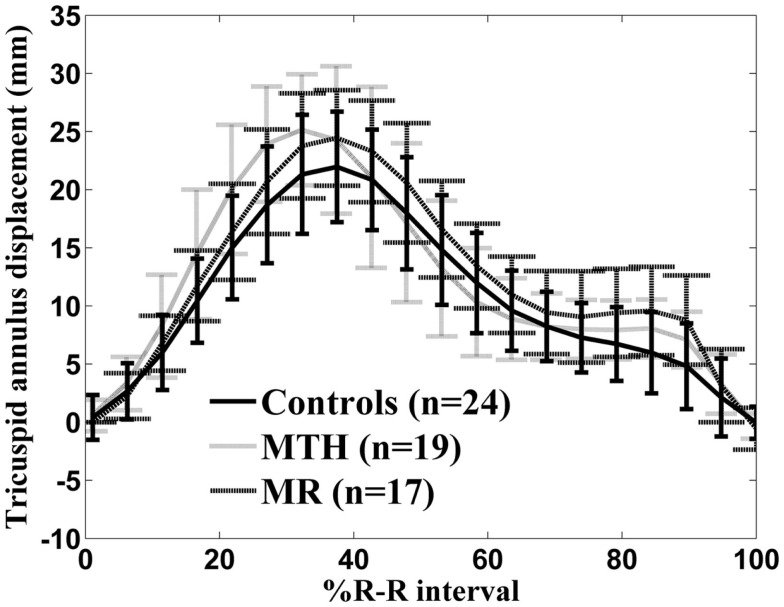
**Tricuspid annulus displacement over time curve for controls (solid line), marathon runners (gray line), and mitral regurgitation patients (dashed line)**. The error bars represent the SD at each measured time point.

Distinct differences were noted in the TA kinetics of MTH compared to controls and MR (Figure 7; Table 3). The peak TA displacement was significantly greater in MTH compared to controls (*p* = 0.0061). Consistent with RVVTC, the TA kinetics demonstrated significantly shorter time to peak displacement in MTH compared to controls. No difference in the TA kinetics of MR compared to controls or MTH was noted except that the peak late diastolic TA velocity was much faster than MTH (*p* = 0.0020) and control (*p* = 0.031) groups (Table 3). This along with somewhat increased atrial filling (Table 1) may indicate impairment of RV diastolic function in MR.

**Table 3:** **Tricuspid annulus motion**.

	Controls	MTH	MR
Peak TA displacement (mm)	22.78 ± 4.59	27.22 ± 4.68*	25.15 ± 4.18
Peak Sys TA velocity (mm/s)	98.41 ± 22.67	113.67 ± 21.05	109.93 ± 23.09
Peak E Dia TA velocity (mm/s)	90.45 ± 28.97	104.38 ± 30.54	105.42 ± 27.54
Peak A Dia TA velocity (mm/s)	66.02 ± 30.04	70.94 ± 33.26	99.33 ± 27.95*^,^^†^
TTP TA displacement (%R–R interval)	38.81 ± 5.87%	34.39 ± 6.19%*	36.57 ± 4.62%

*Values are expressed as mean ± SD*.

*p < 0.05 vs. controls.

*^†^*p* < 0.05 vs. marathon runners*.

*TA, tricuspid annulus; LA, longitudinal; Sys, systolic; E Dia, early diastolic; A Dia, late diastolic; TTP, time to peak*.

## Discussion

Cardiac magnetic resonance is known to yield accurate non-invasive RV volumetric measurements (22–24). In this paper, we examined structural and functional changes in the IVS and right ventricle in response to physiologic and compensated pathologic LV dilation states using standard CMR. Although the proposed modified RV short-axis series (25) can make the RV volume measurements easier and less error-prone, our technique provides a viable means to perform RV volumetric analysis in standard short-axis view and can be applied to clinical practice. RVVTC was derived with correction for TA displacement to provide more consistent systolic and diastolic parameters. Further, accurate characterization of septal and RV geometry and mechanics along with TA kinetics was performed using cine and tagged MRI.

Interventricular septum plays a critical role in the force transmission and maintenance of mechanical performance of biventricular function (4, 26–28). Here, we noted interesting adaptive changes in MTH and MR. The septal circumferential curvature was decreased in both groups compared to controls. This septal flattening is expected and reflects response to increased LV volume overload in MR and a complex interplay of pressure and volume overload on both right and left ventricles in MTH (29). There was no difference in septal principal and maximal strain measurements among the three groups. The preserved septal strains in MR likely reflect an unloading effect of compensated MR and a lack of significant pulmonary hypertension. In contrast, the normal LV geometry and lengthening maintained in MTH allows for preserved IVS strains.

Despite similar RV, EF and RV free-wall strains among the groups, the RVVTC suggests a more optimal systolic and diastolic profile in MTH compared to MR. Consistent with this, parallel changes in the TA kinetics in the three groups were also present. TA kinetics play, an important role in the overall RV systolic and diastolic function (30–32). The enhanced TA kinetics provides a mechanistic rationale for increased RV stroke volume and maintenance of RV systolic function. This finding in the MTH can all be attributed to the preload enhancing effect of increased RV volume.

In conclusion, our study described changes in the IVS mechanics and geometry due to physiologic versus pathologic left ventricle enlargement and its impact on RV function. We found that RV myocardial contractility and local curvature were similar in MTH and isolated compensated MR with preserved LVEF. There were subtle changes in septal mechanics in physiologic LV remodeling compared to controls. This combined with the findings of increased TA displacement reached in a shorter period of time in MTH suggests an overall favorable adaptive RV response in the MTH group.

## Author Contributions

WZ generated all the experimental data for validation and comparison, drew one set of contours for the inter-user variability study, and drafted the manuscript. WF developed the dual-contour propagation algorithm. CS helped in the design of the statistic testing. HG drew one set of contours served as the gold standard for validation, helped in the study design, and the revision of the manuscript. PR helped with manual contouring and study revision. SL identified manual contours to achieve consensus for validation and revised the manuscript. LD participated in study coordination. TD participated in the study design, coordination, and manuscript drafting.

## Conflict of Interest Statement

The authors declare that the research was conducted in the absence of any commercial or financial relationships that could be construed as a potential conflict of interest.

## Supplementary Material

The Supplementary Material for this article can be found online at http://www.frontiersin.org/Journal/10.3389/fcvm.2015.00008/abstract

## Abbreviations

IVS, interventricular septum; MR, mitral regurgitation; MTH, marathon runners; RV, right ventricular; TA, tricuspid annulus; VTC, volume-time curve.
