# Supplementary material for: Improved Right Ventricular Performance with Increased Tricuspid Annular Excursion in Athlete’s Heart
Source: Front Cardiovasc Med. 2015 Apr 30;2:8. doi: 10.3389/fcvm.2015.00008 (PMC4671336; doi:10.3389/fcvm.2015.00008)
Supplement: Supplementary file 1 [file Table_1.PDF]

Supplementary Table SI-1 Differences in RV volumes (expressed as a fraction of RVEDV) computed from propagated and manually-drawn contours

	RV Volume Difference (Fraction of RVEDV)			
	Mean ± SD	99% Confidence Interval		*p value*
Dual - Manual	-0.0085 ± 0.056	-0.037	0.020	0.42
EDNRR - Manual	0.071 ± 0.17	0.044	0.11	<0.0001
ESNRR - Manual	-0.14 ± 0.12	-0.24	-0.17	<0.0001
CAAS MRV ED - Manual	-0.079 ± 0.17	-0.11	-0.051	<0.0001
CAAS MRV ES - Manual	-0.37 ± 0.11	-0.39	-0.34	<0.0001

Dual: dual propagated contours; Manual: manually-drawn contours; EDNRR: contours propagated from manual ED contours using NRR; ESNRR: contours propagated from manual ES contours using NRR; CAAS MRV ED: contours propagated from manual ED contours in CAAS MRV 3.3.1; CAAS MRV ES: contours propagated from manual ES contours in CAAS MRV 3.3.1
