# Supplementary material for: Improved Right Ventricular Performance with Increased Tricuspid Annular Excursion in Athlete’s Heart
Source: Front Cardiovasc Med. 2015 Apr 30;2:8. doi: 10.3389/fcvm.2015.00008 (PMC4671336; doi:10.3389/fcvm.2015.00008)
Supplement: Supplementary file 2 [file Data_Sheet_1.PDF]

APPENDIX

Dual-contour propagation method and tricuspid annulus tracking for RVVTC

Manual contours at RV ED and ES were drawn and propagated using a dual-contour propagation method, which is based on a non-rigid registration (NNR) algorithm (33). Contour propagation, starting from ED and ES contours, was carried out in time throughout the cardiac cycle in each slice. These two sets of propagated contours were then combined via a weighted least-square fit into a single B-spline contour (6). A partial volume summation technique based on automated TA tracking was used to determine the percentage of volume from basal slices to be incorporated in the RV volume computation in each time frame. Excluding the time for manual contouring at ED and ES, the average computation time for dual-propagation was 4.3 minutes for a single study on a 2.61 GHz quad-core computer with 8 GB of RAM whereas manual contouring required approximately 4 hours per study.

In 9 studies, RV contours were manually drawn by consensus of two Level 3 trained CMR specialists on all time frames (HG and SGL). A partial volume summation technique as described above was applied to the basal slices. These contours were used as a gold standard for evaluating and validating the dual-contour propagation algorithm. RV Volumes and VTCs computed from dual-contour propagation, single-propagated contours from ED and ES using NRR, and single-propagated contours from ED and ES using CAAS MRV for Windows Version 3.3.1 (Pie Medical Imaging, Maastricht, The Netherlands) were compared for each study. Comparisons of RV volumes computed from different propagation schemes were performed using mixed modeling via PROC MIXED. To account for the repeated measures within a subject, a compound symmetry correlation structure was assumed. Confidence intervals on the differences based on the fitted mixed model were constructed each at a 99% level to achieve a joint confidence level of at least 95% for this set of confidence intervals using Bonferroni adjustment (34).

Comparison between contour propagation methods

No significant differences were found in RV volumes from the dual-propagation and manually-drawn contours as depicted in **Table SI-1**. In comparison, NRR from ED overestimated RV volumes; NRR from ES underestimated RV volumes; CAAS MRV ED and CAAS MRV ES underestimated RV volumes.

The VTCs for 9 controls computed from the dual-propagated and manually-drawn contours were shown in Supplementary **Figure SI-1**. Dual propagated VTCs showed good agreement with the manual VTCs, suggesting that contours manually drawn at ED and ES were consistently propagated to the other time frames in the cine sequence.

Inter-user variability

To assess inter-user variability in volumes computed from propagated contours, two sets of RV endocardial contours were manually drawn in each study at ED and ES by different users, a Level 3 trained CMR specialist (HG) and a user with 4 years of experience (WZ). Each set of ED

and ES contours was propagated using the dual contour technique, and VTCs and RV ejection/filling rates were computed.

No significant differences were observed between RVEDV (User2-User1: 2.38±4.64, p=0.16), RVESV (User2-User1: -1.33±5.30, p=0.47), RVEF (User2-User1:0.14±2.66, p=0.88), RVPER (User2-User1: -0.06±0.28, p=0.56), RVePFR (User2-User1: -0.06±0.39, p=0.66), and RVaPFR (User2-User1: 0.20±0.34, p=0.12) values computed from contours propagated with ED and ES contours drawn by two different users. RVEDV (ρ=0.99, p<0.0001), RVESV (ρ=0.96, p<0.0001), RVEF (ρ=0.94, p=0.0021), and RVPER (ρ=0.88, p=0.0017) were highly correlated between the two users. Supplementary **Figure SI-2** and **SI-3** show the scatter plots and Bland-Altman plots comparing measurements of RVEDV, RVESV, RVEF, RVPER, RVePFR, and RVaPFR between the two users.
