# Supplementary figures and images for: Improved Right Ventricular Performance with Increased Tricuspid Annular Excursion in Athlete’s Heart
Source: Front Cardiovasc Med. 2015 Apr 30;2:8. doi: 10.3389/fcvm.2015.00008 (PMC4671336; doi:10.3389/fcvm.2015.00008)

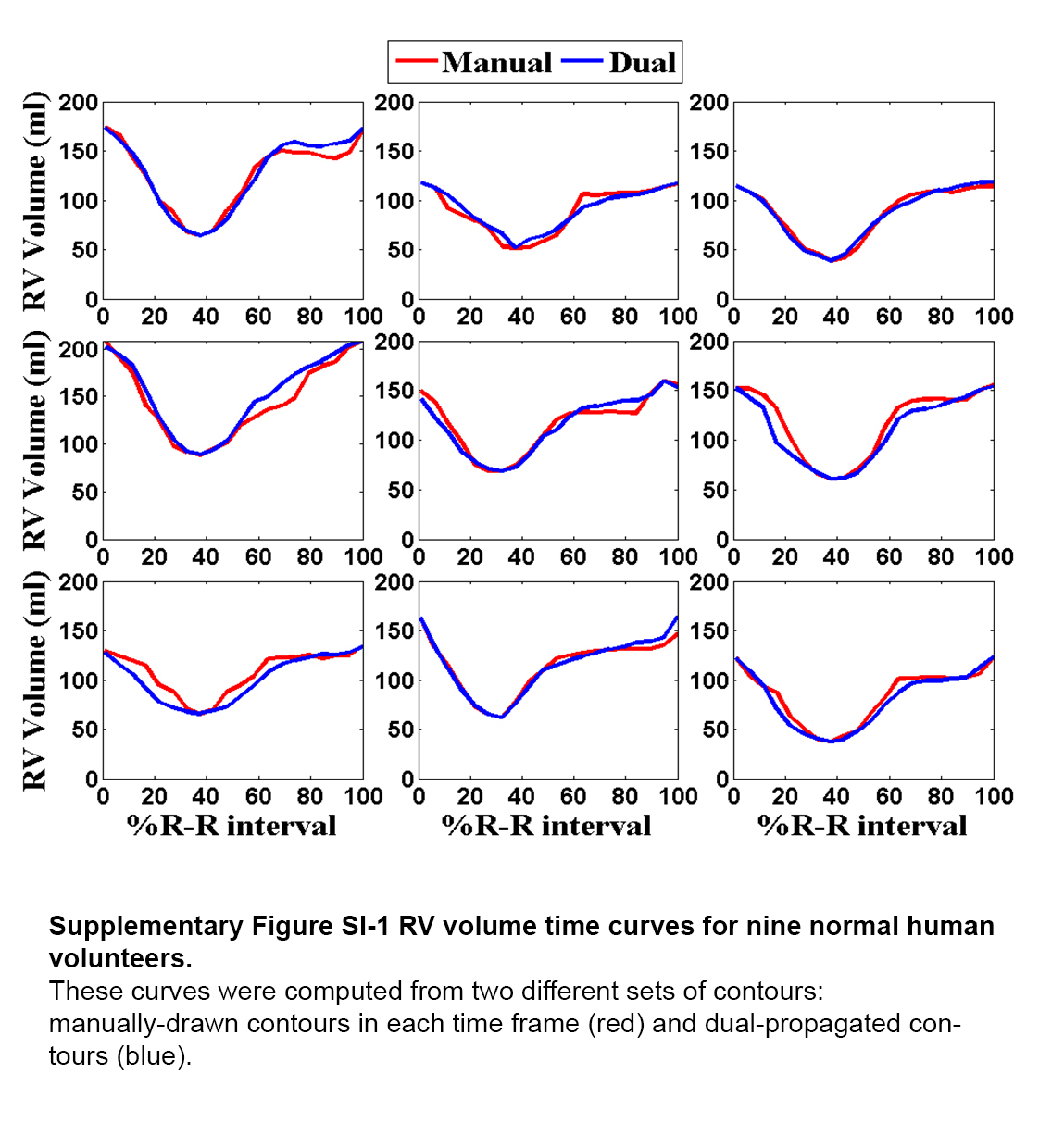

Supplement: Supplementary file 3 [file Image_1.JPEG]

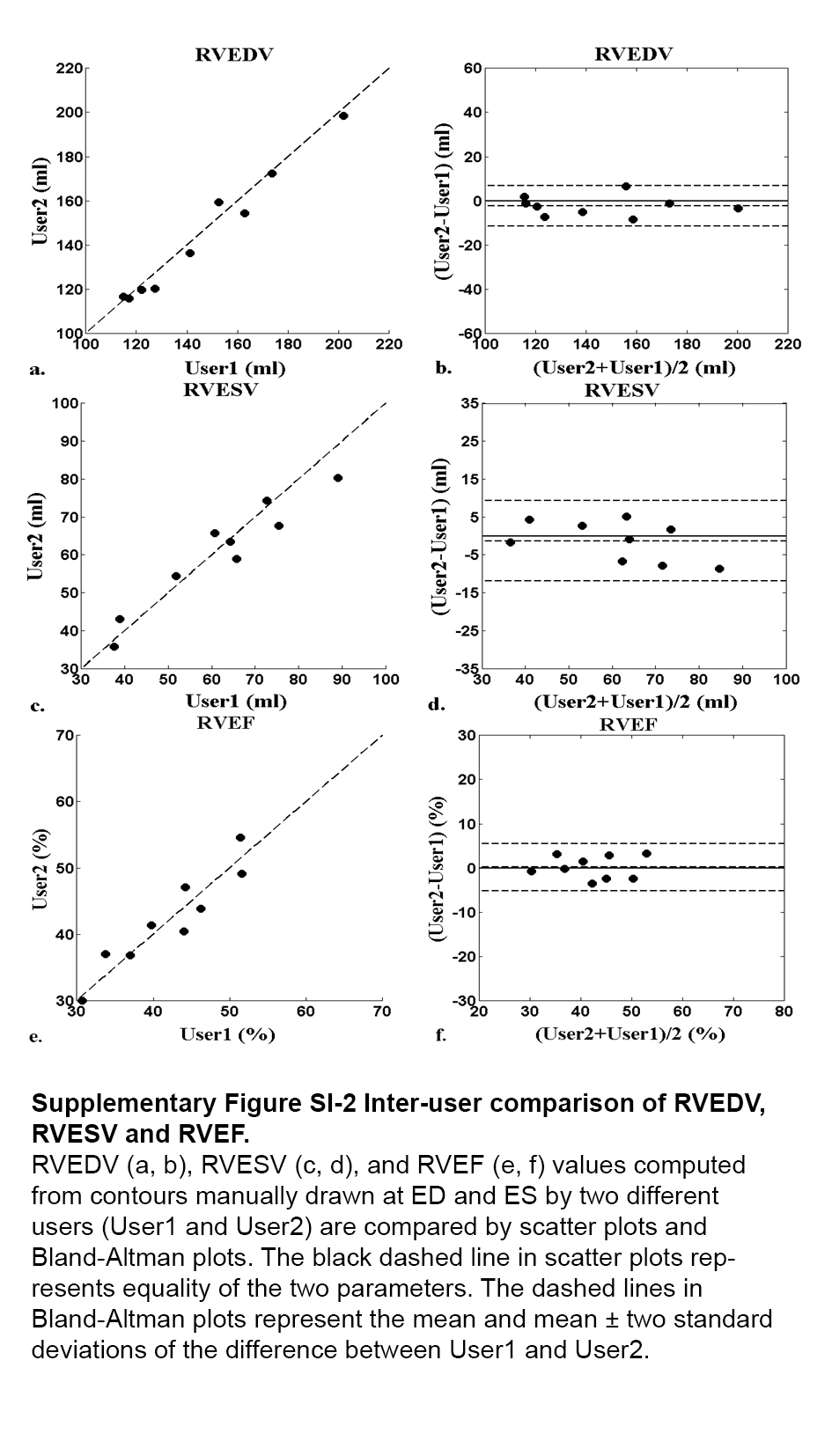

Supplement: Supplementary file 4 [file Image_2.JPEG]

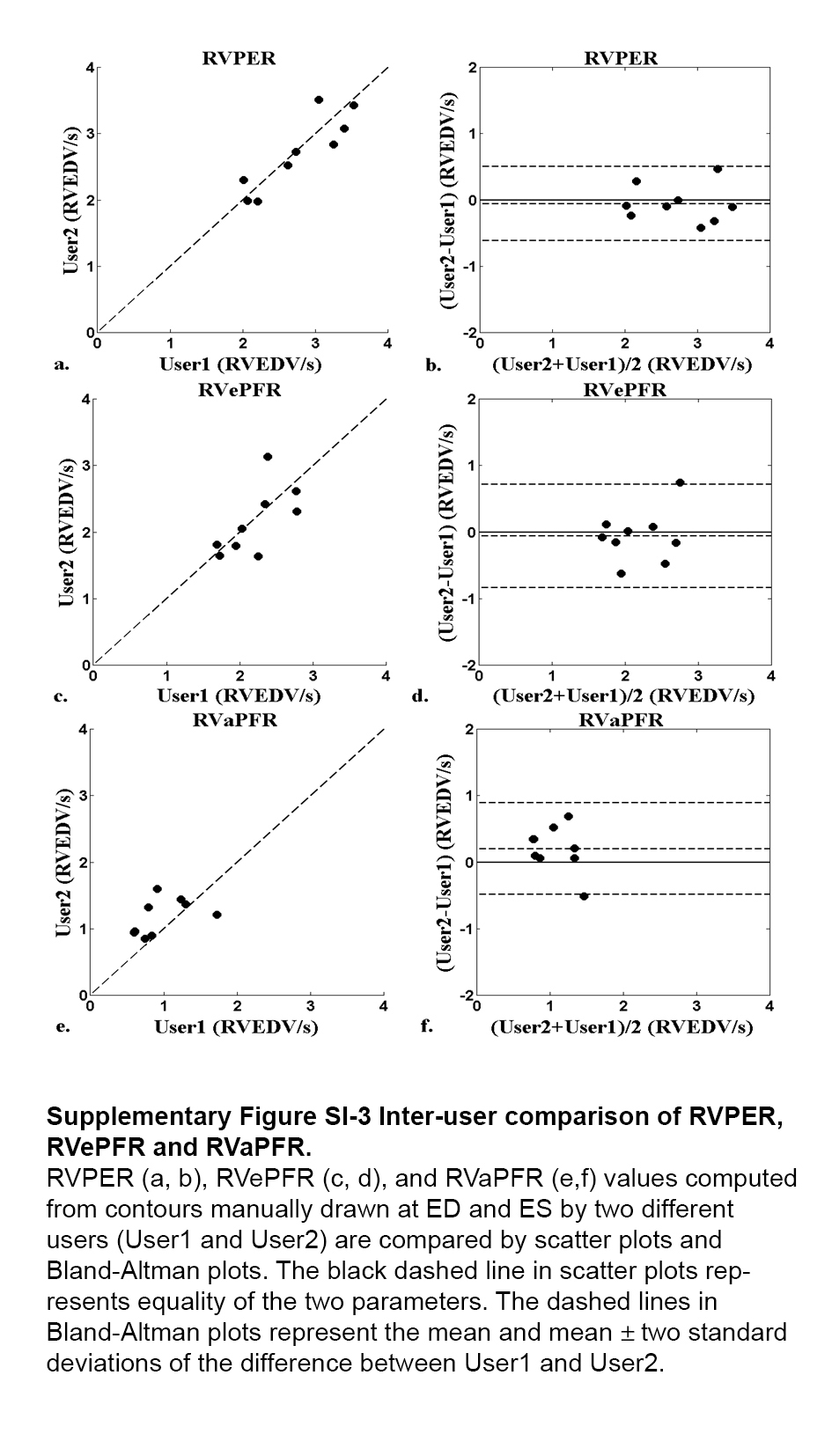

Supplement: Supplementary file 5 [file Image_3.JPEG]
